# Effectiveness of Jena DM® Herbal Formulation as Complementary Therapy to Conventional Oral Hypoglycemic Agents in Type-2 Diabetes Mellitus: A Quasi-experimental Study

**DOI:** 10.7759/cureus.62649

**Published:** 2024-06-18

**Authors:** Joshua Kiptoo, Tadele Mekuriya Yadesa, Clement Olusoji Ajayi, Oliver Kushemererwa, Adellah Kantengwa, Anthony Muyingo

**Affiliations:** 1 Pharmacy, Mbarara University of Science and Technology, Mbarara, UGA; 2 Pharmacy, Kampala International University, Ishaka, UGA; 3 Diabetes & Endocrinology, Mbarara Regional Referral Hospital, Mbarara, UGA; 4 Internal Medicine, Mbarara University of Science and Technology, Mbarara, UGA

**Keywords:** jena dm®, sesquiterpene lactones, herbal medicine, weight loss, insulin resistance, glycated hemoglobin, artemisia annua

## Abstract

Background:* *There is vast preclinical evidence that indicates that extracts from several Artemisia plant species have significant antidiabetic benefits. However, clinical evidence is limited to this effect.

Objective: We sought to evaluate the effectiveness of *Jena DM^®^* (an *Artemisia annua*-based poly-herbal formulation) on glycemic control (Hb A1C) and insulin metabolism (HOMA), when administered as a complementary therapy in type-2 diabetes mellitus (T2DM). This study was supported by a research grant (JRD005) from Jena Herbals (U) Ltd, which is a local herbal medicines manufacturing facility in Uganda.

Methods: We conducted a 12-week quasi-experimental study, involving 118 patients under routine follow-up at a diabetes and endocrinology clinic. Random assignment to either conventional or experimental study groups was done using a random number generator (Microsoft Excel version 16.0). Participant sociodemographic and clinical data as well as whole blood samples (3-5 mL) were obtained at scheduled clinic visits. Medication adherence was assessed using the Hill-Bone Scale, and adverse drug events (ADEs) using the Naranjo causality and the National Institute of Allergy and Infectious Diseases, Division of AIDS (DAIDS) scales. Group differences in glycemic control (HbA1C), fasting serum insulin (FSI) indices (% HOMA2-B, HOMA-IR), and other cardiometabolic parameters were assessed using independent samples t-test, and Pearson chi-square statistical tests were used. A p-value <0.05 was considered statistically significant. Ethical approvals were obtained before the study commencement.

Results: 12-week daily complementary therapy with *Jena DM^® ^*showed no significant effect on Hb A1C reduction (0.1 (95% CI: -0.56, 0.80) %; *p*=0.798); however, we observed a significant reduction in total body weight (2.0 (95% CI: 0.73, 3.28) kg; *p*=0.002). The overall frequency of self-reported ADEs including dizziness was significantly higher among patients that used *Jena DM^® ^*(p=0.001). Epigastric pain was the most severe ADE necessitating clinical management. There was no significant difference in the homeostatic model assessment for insulin resistance (HOMA2-IR) between study groups.

Conclusion: In contrast to a few studies that previously showed significant hypoglycemic effects of *Artemisia-based* extracts, this study did not show a statistically significant reduction on HbA1C during a 12-week complementary use of *Jena DM^® ^*in patients with T2DM. Based on the findings of this study, future research should evaluate the long-term effects of *Jena DM^® ^*on body weight, overall insulin metabolism, and the subsequent effect on glycemic control in T2DM.

## Introduction

Type-2 diabetes mellitus (DM) is a complex metabolic disorder, that is characterized by progressive loss of pancreatic β-cell secretory function and insulin receptor sensitivity [1]. The burden of type-2 DM is among the top leading public health concerns [2]. Due to their perceived lower cost, higher accessibility, and comparably better safety profile, scientific interest in antidiabetic remedies of herbal origin has tremendously increased in the recent past [3-5].

Herbal medicines are defined as ‘finished labeled medicinal products containing active ingredients of the plant or other plant material or combinations’ [6]. Several medicinal plants including aloe vera, psyllium fiber, fenugreek seeds, *Nigella sativa*, and the Chinese antidiabetic formula Jinqi Jiangtang are known to reduce HbA1c. Several culinary food additives from the Artemisia plant spp. have also emerged as novel antidiabetic therapeutic targets among patients in type-2 DM, including *Artemisia dracunculus* [7-8]. However, clinical evidence on the anti-hyperglycemic effects of *Artemisia annua* extracts remains scanty despite *A. annua* being the most abundant Artemisia plant spp. in the tropics, and among the six Artemisia plant spp. with the highest percentage of sesquiterpene lactones (SLns) [9].

SLns are a lipophilic sub-group of terpenoids characterized by a structural isoprenoid (lactone) ring system [10]. Artemisia-derived SLns are known to activate the insulin receptor substrate (IRS) and enhance phosphorylation of protein kinase B in the skeletal muscle of animal models, thus leading to improved insulin sensitivity [11]. Other relevant cardio-metabolic effects attributed to Artemisia-derived SLns include blood pressure reduction, direct glycemic control, modulation of insulin and leptin signaling via protein tyrosine phosphatase inhibitor 1B (PTP1B) inhibition [12], inhibition of α-glucosidase, attenuation of NF-κB (nuclear factor-kappa B) signaling pathway in insulin resistance, restoration of pancreatic beta cell secretory function, and regulation of adipocyte production and pancreatic alpha-beta cell trans-differentiation [7]. All these mechanisms synergistically influence the physio-pathological pathway of type-2 DM.

Generally, there is a paucity of evidence regarding the clinical efficacy and safety of herbal preparations with *A. annua* extracts. Therefore, we sought to determine the effectiveness of an *A. annua*-based herbal formulation (*Jena DM®*) as a complementary therapy with conventional oral hypoglycemic drugs in type-2 DM.

## Materials and methods

Study design, period, and setting

We conducted a quasi-experimental study over a 12-week period, at the ambulatory Diabetes and Endocrinology Clinic of Mbarara Regional Referral & Teaching Hospital (MRRH). MRRH is a tertiary-level public hospital in south-western Uganda. The hospital has both inpatient and outpatient care services, including the diabetes and endocrinology (D&E) clinic. The D&E clinic runs once every week and is headed by a consultant diabetology physician, with the support of the clinic nurses and dispensers. On the routine days, residents of internal medicine rotating in the clinic provide the medical services with referral to other clinics or inpatient units as necessary. On average, about 150 patients visit the clinic weekly.

Study participants

Patients on follow-up routine care for type 2 DM were consecutively screened for eligibility. We included patients aged ≥ 30 years, with a clinical diagnosis of type-2 DM, and taking at least one oral hypoglycemic agent (OHA). Patients using insulin-based regimens, and those with a clinical history of liver injury were excluded from the study.

Study groups

Patients were assigned to either study groups, depending on whether or not, they were using *Jena DM®* as a complementary therapy at the time of enrolment. The conventional (control) group comprised participants treated with metformin, with or without a sulfonylurea, while the experimental group comprised participants treated with metformin, with or without a sulfonylurea, in addition to *Jena DM®* herbal extract.

Description of *Jena DM®*

*Jena DM®* is a poly-herbal formulation packaged as dry green pellets obtained from three tropical plants. De-artemisinized *A. annua* is the major ingredient in *Jena DM®* (Table 1). *Jena DM®* is licensed as a complementary therapy for the management of type-2 DM and prediabetes in Uganda (registration number: THA778) [13]. The formulation is available in drug outlets across Uganda.

**Table 1:** Composition of Jena DM®

Name of the Herb		Plant part(s) used	Quantity per teaspoonful (2.7-3 g)
Botanical	English local		
De-artemisinized *Artemisia annua* L. (Asteraceae)	African wormwood	Leaves	1.00 g
*Cymbopogon citratus* Stapf. (Poaceae)	Lemon grass	Leaves	0.25 g
*Persea americana *Mill. (Lauraceae)	Avocado	Seed	1.50 g

Experiment procedure

Ethical approval was obtained from the Mbarara University of Science and Technology Research Ethics Committee (MUST-2021-283). This study was conducted in accordance with the declaration of Helsinki. First, written informed consent was obtained from all eligible participants at the clinic triage. A standardized questionnaire was used to capture the relevant participant data. A bi-weekly clinical assessment was done by the study physician to screen and monitor suspected adverse drug events (ADEs). Naranjo ADE scale was used to establish the causality between the medications and the ADEs. The severity of the ADEs was rated using the National Institute of Allergy and Infectious Diseases (NIAIDS), Division of AIDS (DAIDS) criteria. At each scheduled clinic visit, the study pharmacist assessed medication adherence using the Hill-Bone medication adherence scale and provided medication counseling service where necessary. Additionally, vital measurements, including blood pressure, height, body weight, and fasting blood sugar were measured by the study nurse. About 5 mLs of venous blood that was obtained from each participant and kept in a vacutainer containing 0.1% ethylenediaminetetraacetic acid (EDTA) was used to determine glycemic control (determined by glycated hemoglobin (Hb A1C)) and fasting serum insulin (FSI) at baseline and endline. All collected blood samples were transported in an ice-cold carrier (-20 0C) to the MRRH laboratory. Plasma for measurement of FSI was subjected to continuous freeze-thawing at -20 0C for 6 weeks when the determination of serum insulin levels was done. Serum was obtained by centrifuging plasma samples at a speed of 1,500 x g for 30 minutes at 4°C. Fasting insulin and fasting glucose values were used to determine HOMA2-IR. All participants received their routine oral hypoglycemic drugs at the clinic. All participant’s data were assessed for completeness and accuracy by the study investigators before storage in a secure site. 

Study outcome measures

The primary outcome was the 12-week mean change in the Hb A1c. The 12-week duration was used based on the clinical rational of red blood cells turnover rate. We hypothesized that *Jena DM®* would significantly improve HbA1c in T2DM. The secondary outcomes included the proportion of participants with significant self-reported ADEs, mean change in body weight, and HOMA2-IR.

Quality control

A calibrated weighing scale was used to measure body weight. All blood samples were centrifuged and assayed in random order in the same analytical run to reduce systematic bias and inter-assay variation. MRRH laboratory is ISO-certified, and all samples for analysis were run in triplicates. Test results of the same samples at MRRH are periodically compared with results from the Lancet Lab a Level V ISO-certified laboratories.

Statistical analysis

Data was analyzed using the Social Package for Statistical Sciences (SPSS version 21, Inc., Chicago, IL, USA) software. Normality was tested using Levene’s test. The within-group comparison was done using a paired samples t-test. A two-tailed chi-square and independent samples t-tests were used to determine between-group differences. Data was presented as mean(±SD) and median (IQR). Self-reported adverse effects (AEs) were summarized as frequencies, and data was presented in bar charts. A p-value less than 0.05 was considered statistically significant.

## Results

Enrolment procedure

**Figure 1:**
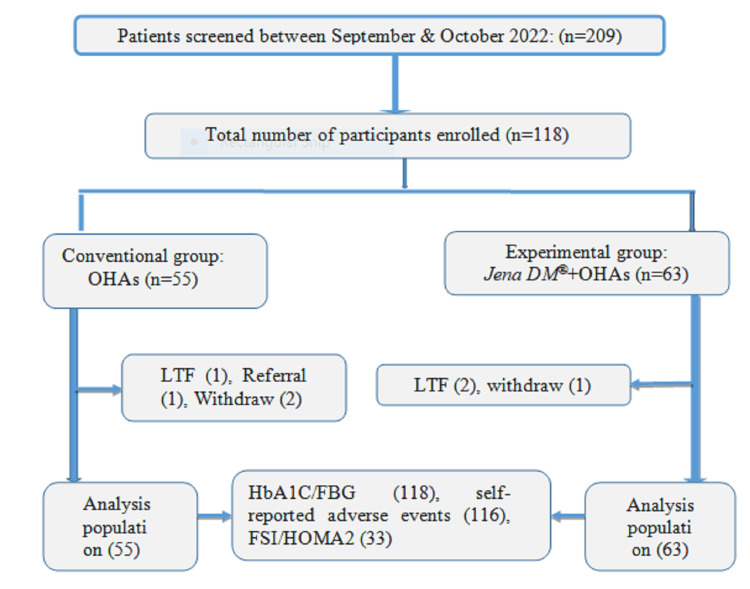
Participant enrolment and group allocation FBG: fasting blood glucose; HbA1C: glycated hemoglobin; LTF: lost to follow-up; OHA: oral hypoglycemic agents

A total of 209 patients with type-2 DM were screened for eligibility and 118 participants fulfilled the eligibility criteria and were enrolled in the study. After 12 weeks of follow-up, 116 participants (98.3%) remained in the study and were considered for statistical analysis (Figure 1).

Comparison of baseline participant characteristics: Overall, the participant’s mean age was 58.9±12.1 years, and about three-quarters (88/118) were female. Study participants were generally overweight (BMI 29.6±5.8) and had poor adherence to prescribed medications, and uncontrolled glycemia (HbA1c of 8.4±1.8%). At baseline, there was no statistically significant difference between the two study groups (Tables 2-3).

**Table 2:** Comparison of participant baseline sociodemographic and clinical characteristics BMI: body mass index; DM: diabetes mellitus; FBG: fasting blood glucose; HbA1c: glycated hemoglobin; SBP: systolic blood pressure; SD: standard deviation

Variables	Overall (N=118), mean±SD	Conventional (n=55), mean±SD	Experimental (n=63), mean±SD	p-value
Age	58.9±12.1	59.8±13.0	58.2±11.3	0.484
Gender (female)	88 (75%)	41	47	0.994
Duration of type-2 DM (years)	6.7±6.2	7.4±7.2	6.4±5.2	0.368
Medication adherence (Good)	41 (34.7%)	14	27	0.086
Metformin dose (g/day)	1.5±0.6	1.6±0.6	1.5±0.6	0.159
Glibenclamide dose (mg/day)	5.3±1.8	5.2±1.4	5.4±2.2	0.595
FBG (mMol/L)	9.8±4.1	10.1±4.3	9.8±3.9	0.861
Hypertension (n, %)	77, 65.3%	39	38	0.214
HbA1C (%)	8.4±1.8	8.4±1.7	8.6±1.9	0.619
SBP (mmHg)	143.5±21.2	144.1±20.9	144.3±21.6	0.666
Body weight (Kg)	72.8±14.9	73.3±15.2	72.5±14.8	0.777
BMI (Kg/m^2^)	29.6±5.8	29.8±6.2	29.4±5.5	0.735

**Table 3:** Comparison of participant baseline insulin metabolic profiles and indices FSI: fasting serum insulin; HOMA2-IR: homeostatic model assessment for insulin resistance; HOMA2-B: homeostatic model assessment beta; SD: standard deviation

Variables	Overall (N=45), mean±SD	Conventional (n=20), mean±SD	Experimental (n=25), mean±SD	p-value
FSI (pMol/L)	59.6±34.1	48.77±34.26	73.24±36.398	0.305
HOMA2-B (%)	39.2±36.5	32.92±27.75	37.25±25.51	0.337
HOMA2-IR	1.5±1.4	1.06±0.69	2.11±1.88	0.168

Effectiveness of *Jena DM®*

**Figure 2:**
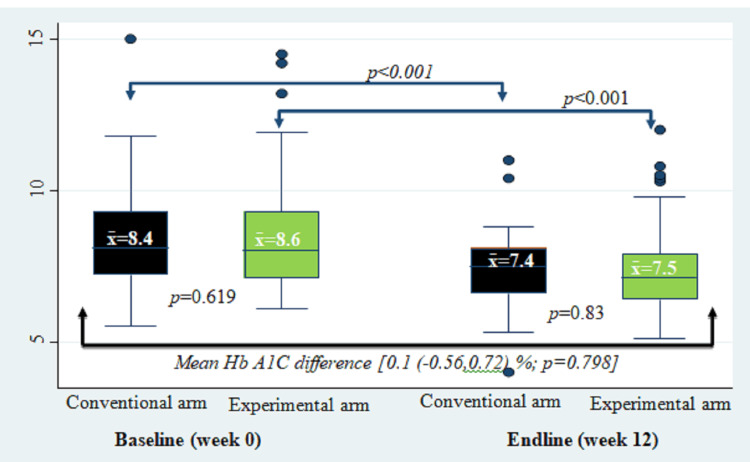
Mean change in glycated hemoglobin concentration HbA1C: glycated hemoglobin *p-value calculated by student t-test.

There was no statistically significant difference in Hb A1c, HOMA2-B, and HOMA2-IR mean changes between study groups (Tables 4-5; Figure 2).

**Table 4:** Comparison of participant clinical characteristics after 12 weeks *Statistically significant mean difference BMI: body mass index; CI: confidence interval; FBG: fasting blood glucose; HbA1c: glycated hemoglobin; SBP: systolic blood pressure

Variables	Conventional (n=55), mean difference (95% CI)	Experimental (n=63), mean difference (95% CI)	Between-group mean difference (p-value)
HbA1c (%)	-1.0 (-1.5, -0.5)	-1.1 (-1.6, -0.8)	0.1 (0.798)
FBG (mMol/L)	-1.6 (-2.8, -0.5)	-1.7 (-2.5, -0.7)	-0.01 (0.928)
Body weight (Kg)	1.6 (0.5, 2.7)	-0.4 (-1.2, 0.4)	2.0* (0.002)
BMI (Kg/m^2^)	0.6 (0.2, 1.0)	-0.2 (-0.5, 0.1)	0.8* (0.002)
SBP (mmHg)	-2.1 (-7.5, 3.4)	-4.3 (-8.5, -2.1)	2.2 (0.514)

**Table 5:** Comparison of participant insulin metabolic profiles and indices after 12 weeks CI: confidence interval; FSI: fasting serum insulin; HOMA2-IR: homeostatic model assessment for insulin resistance; HOMA2-B: homeostatic model assessment beta

Variables	Conventional (n=12), mean difference (95% CI)	Experimental (n=21), mean difference (95% CI)	Between-group mean difference (p-value)
FSI (pMol)	22.6 (-21.0, 66.1)	1.9 (-18.9, 22.6)	20.7 (0.305)
HOMA2-B (%)	17.0 (-3.1, 37.0)	6.5 (-6.6, 19.5)	10.5 (0.337)
HOMA2-IR	0.4 (-0.4, 1.2)	-0.5 (-1.4, 0.4)	0.9 (0.168)

**Figure 3:**
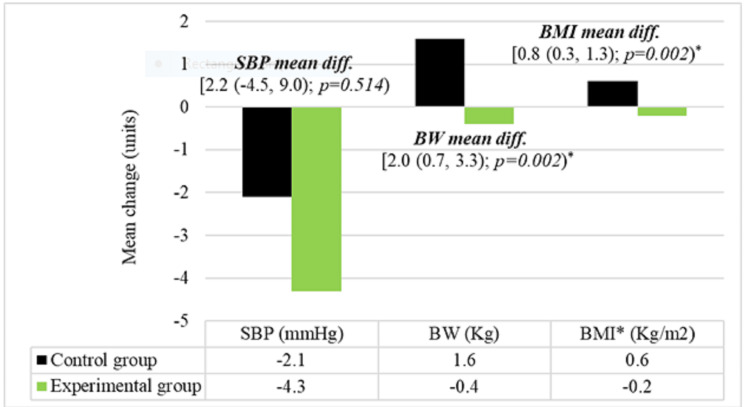
Comparison of change in mean blood pressure, body weight, and BMI SBP: systolic blood pressure, BW: body weight, BMI: body mass index *Statistically significant mean difference value (p < 0.05).

At endline, there was a statistically significant reduction in body weight and BMI in the experimental group compared to participants in the conventional study group (p=0.002). Mean differences in systolic blood pressure reduction were not significant between the two groups (Figure 3).

Self-reported ADEs

**Figure 4:**
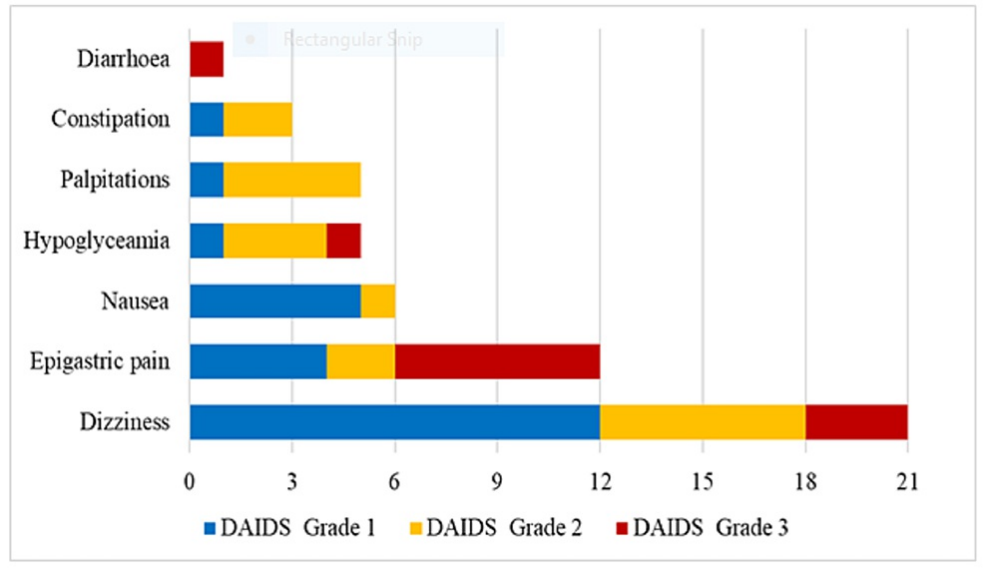
Severity of self-reported adverse drug events DAIDS: Division of AIDS criteria for adverse drug event grading

Out of the 116 study participants, 53 (45.7%) self-reported at least 1 (0.46±0.50) ADE. Overall, the number of self-reported ADEs was significantly higher in the experimental group (37 vs. 16; p= 0.001). Dizziness was the most self-reported ADE (21, 39.6%), with a significantly higher frequency in the experimental group (p=0.021) (Table 4). The majority (24, 45.2%) of self-reported ADEs were mild in severity (Grade 1). However, epigastric pain was the most self-reported severe (Grade 3) ADE (6, 54.5%) (Figure 4).

**Table 6:** Self-reported adverse drug events (ADEs) among study participants *statistically significant difference

Reported AEs	Frequency of AEs (n, %)	Study group		Pearson chi-square/Fisher’s exact test (p-value)
		Conventional group (n=51)	Experimental group (n=60)	
Dizziness	21 (39.6%)	5	16	0.021^*^
Epigastric pain	12 (22.6%)	4	8	0.255
Nausea	6 (11.3%)	2	4	0.408
Hypoglycemia	5 (9.4%)	3	2	0.434
Palpitations	5 (9.4%)	1	4	0.228
Constipation	3 (5.7%)	1	2	0.552
Diarrhea	1 (2.0%)	0	1	0.534
Total AEs	53 (100.0%)	16	37	0.001^*^

## Discussion

This study evaluated the effectiveness of a de-artemisinized *A. annua*-based poly-herbal formulation, *Jena DM®*, as complementary anti-hyperglycemic therapy among patients with type-2 DM. Overall, the 12-week mean change in Hb A1c was statistically insignificant between study groups. Participants on the experimental arm experienced a higher frequency of ADEs (especially dizziness) and had a significantly lower mean body weight at the end of the study.

Persistent uncontrolled glycemia is the hallmark of type-2 DM and a primary driver of diabetes progression and disease-related complications. Despite the presence of effective pharmaceutical products for the management of diabetes, uncontrolled glycemia remains prevalent in low and middle income countries [14-15]. The pharmacologic effects of *A. annua* extracts on various diabetes biomarkers have been extensively explored using animal models [11]. However, this study did not demonstrate a statistically significant difference in 12-week glycemic control between study groups. In their review, Sasani et al. concluded that complementary therapy with different Artemisia extracts significantly improved insulin resistance (HOMA2-IR), but benefits in glycemic control remained unclear [16]. Extracts of Artemisia spp have diverse secondary metabolites [17], and the majority of the known potent anti-hyperglycemic phytochemical compounds, i.e., flavonoids, coumarins, and essential oils, are largely abundant in a few Artemisia spps like *A. drancunculus* and *A. princeps* [18].

In this study, participants using *Jena DM®* as complementary therapy had significantly higher self-reports of ADEs (p=0.001), especially dizziness (p=0.021). Dizziness may be attributed to the additive hypoglycemic effect of *Jena DM®* and OHA in the experimental group. Notably, epigastric pain was the most reported severe ADE, that necessitated dose modification of *Jena DM®*, clinical management of acid disorders, or both. As observed with the study participants, diabetes mellitus, poor glycemic control, and use of metformin pose a significant inherent risk for functional gastrointestinal disorders in type-2 DM [19, 20]. However, in this study, epigastric pain can partly be attributed to the physiochemical properties of *Jena DM®*. Depending on the quantity and method of extraction, extracts of *Cymbopogon ​​​​​*​*citratus* are known to have variable degrees of acidity, with potential consequences on gastrointestinal health [21]. Patients on *Jena DM®* should be monitored for epigastric pain and active post-market surveillance should be done to establish its causality in the wider population.

Therapies with significant weight loss have been associated with significant metabolic benefits, including glycemic control [22], improvement in pancreatic beta cell function [23], and significant risk reduction for type-2 DM disease progression [24]. This study reported significant weight loss in participants on complementary therapy with *Jena DM®*. Animal models have described the positive role of terpenoids in overall energy intake, body weight, and regulation of free fatty acid metabolism via adipocyte peroxisome proliferator-activated receptor (PPAR) gene expression [23]. Obesity is characterized by systematic and low-grade chronic inflammation [25], consequent hypoxia, and hypertrophy of adipocytes. Due to their potent immunosuppressive effects [26], artemisinin derivatives may induce weight loss by ameliorating complications of obesity-related chronic systemic low-grade inflammation [27] and enhancing the metabolic transformation of white fat [28]. These mechanisms may plausibly explain the concurrent effects of the *Jena DM®* extracts on both insulin resistance and body weight. Although a few Artemisia spp have demonstrated significant clinical effects on insulin resistance [16], the only evidence on *A. annua* has shown improvement in insulin resistance by enhancing peripheral glucose transporter type 4 (GLUT-4) receptor expression and modulating adipocyte adiponectin/leptin metabolism is in animals [29]. This study did not demonstrate a significant effect on reduction in insulin resistance. Second, human and in-silico studies have reported anti-hypertensive effects of Artemisia spp extracts, primarily through angiotensin converting enzyme 1 (ACE-1) inhibition [7, 30]. However, this study found weak evidence to this end. Future studies should assess this effect given its benefits in diabetic kidney disease prevention.

Without randomization and blinding, this study might have incurred some confounding and selection bias that can affect the interpretation of the findings. This study did not employ a placebo in the conventional group. Without a placebo, it is difficult to distinguish the effect of the intervention and the placebo on the primary outcome. The study was not statistically powered to detect differences in secondary outcome measurements, yet there were larger absolute differences between groups. This may have obscured the true difference in study outcomes. Assessment of clinical safety based on self-reports rather than laboratory-based tests may subject our findings to recall and desirability biases. Lastly, the insulin levels reported in this study were measured at single time points, rather than the recommended average of three measurements done at 5-minute intervals. The serum insulin levels may vary within the morning hours of sample collection.

## Conclusions

A 12-week daily complementary therapy with *Jena DM®* did not show a significant effect on glycemic control, but showed a statistically significant weight reduction and therefore may be of interest in the management of type-2 DM. In the future, randomized controlled trials with a longer participant follow-up duration are necessary to ascertain the overall effectiveness of *Jena DM®* on glycemic control, and insulin metabolism among patients with type-2 DM.
